# Allelopathic effects of *Thuidium kanedae* on four urban spontaneous plants

**DOI:** 10.1038/s41598-024-65660-7

**Published:** 2024-06-26

**Authors:** Muyan Xie, Xiurong Wang

**Affiliations:** College of Forestry, Guizhou University, Guiyang, 550025 Guizhou China

**Keywords:** Plant sciences, Ecology, Environmental sciences

## Abstract

The spontaneous plant landscape is a key focus in the development of urban environments. While many spontaneous plants can coexist with bryophytes to create appealing wilderness landscapes, the potential allelopathic effects of bryophytes on the growth of neighboring spontaneous plants remain uncertain. This study evaluated the allelopathic impact of *Thuidium kanedae* aqueous extracts on the germination and seedling growth of prevalent urban spontaneous plants by analyzing seed germination, seedling growth morphology, and associated indices. We also investigated the allelopathic potential of the predominant compounds in the extract on seed germination. Our findings reveal that the aqueous extract significantly impeded the seed germination of *Ophiopogon japonicus*, *Taraxacum mongolicum*, and *Viola philippica*, with the level of inhibition correlating positively with concentration. In contrast, *Senecio scandens* seed germination showed a concentration-dependent reaction, with low concentrations promoting and high concentrations hindering germination. The extract consistently reduced root length in all four species, yet it appeared to increase root vigor. The chlorophyll content in *O. japonicus* and *V. philippica* seedlings reached a maximum at a concentration of 5 g/L and decreased with higher extract concentrations. The treatment resulted in elevated catalase and soluble protein levels in the seedlings, indicating that the extract induced stress and enhanced the stress resistance index. L-phenylalanine and 2-phenylethanol, substances present in the extract, were notably inhibitory to seed germination across all species, except for *O. japonicus*. Notably, 2-phenylethanol exhibited a stronger allelopathic effect than L-phenylalanine. Allelopathy synthetical effect evaluation showed that high concentration of aqueous extract allelopathic inhibition effect on seed germination of four plant species, but allelopathic promotion effect on physiological and biochemical growth of *Taraxacum mongolicum*, *Senecio scandens* and *Viola philippica*. In summary, the study demonstrates that bryophytes exert allelopathic effects on neighboring spontaneous plants, with the degree of influence varying among species. This suggests that the germination and growth of spontaneous plant seeds may be selective in bryophyte-dominated habitats and that the density of bryophytes could shape the evolution of these landscapes.

## Introduction

In the face of climate change and ecological degradation due to rapid urbanization, the cultivation of plant landscapes in urban green spaces has resulted in issues such as excessive resource consumption and a lack of diversity in the landscape. With the growing public awareness of ecological conservation, spontaneous plants that can thrive in urban environments, possess ornamental value, and provide ecological benefits have attracted significant attention^1^. Spontaneous plants demonstrate traits of robust adaptability, low maintenance requirements, and wide distribution^2^, making them a crucial element of urban plant diversity and essential for establishing sustainable, low-maintenance plant landscapes^3,4^. Despite their significance, native self-dispersed species are underutilized in urban greening initiatives. Research on spontaneous plants mainly focuses on species identification, distribution, succession patterns, potential applications, and public perception^5–8^, with limited exploration of plant interactions within the context spontaneous plants and their application in urban landscaping. In more natural urban landscapes, bryophytes often coexist harmoniously with indigenous herbs or bottom layer plants, creating a wilderness aesthetic that promotes ecosystem stability and ecological benefits. Investigating the impact of bryophytes on the growth of neighboring plants and understanding the mechanisms of their allelopathic effects are crucial for the planning and management of urban wilderness landscapes.

Allelopathy refers to the biological phenomenon where plants secrete specific chemicals into their environment, influencing the growth of neighboring flora through either promotion or inhibition^9,10^. Plants enhance their competitiveness through allelopathy^11^, thereby shaping community composition, succession, and the spatial distribution of species within ecosystems^12,13^. Bryophytes play essential roles as primary productivity contributors in forests and various ecosystems, often engaging in interactions with other plant species within the community. Due to their lack of true roots, bryophytes are ideal model plants for allelopathy research^14^. Current studies on bryophytes allelopathy primarily focus on its impact on crop growth or invasive plant species^15,16^, categorized based on the tested materials. These categories include basic tests that utilize crops and vegetable seeds as bioassay materials and exploratory experiments that concentrate on herbaceous plants sharing symbiotic or competitive relationships with bryophytes^17–19^. Despite the widespread recognition of bryophytes' allelopathic effects, certain research gaps persist, including inadequate investigation into the isolation, identification, and mechanisms of the key components responsible for the allelopathic effects of specific bryophytes, limited exploration of plant selection for moss landscape construction, scarce studies examining the impacts on the growth of neighboring symbiotic spontaneous, and insufficient reports on allelopathic interactions between mosses and native self-dispersed species.

Bryophytes and bottom layer plants are common in urban green space in karst landform areas, where a natural wilderness landscape can spontaneously form by sowing various native self-dispersed plant species seeds. The key factor in creating such landscapes lies in the germination of seeds among bryophytes and whether their growth is influenced by neighboring bryophytes' allelopathy. In our study, we focused on four spontaneous plant species known for their ornamental value and wide distribution, in addition to common dominant bryophyte species. Extracts of allelochemical from *Thuidium kanedae* were obtained using distilled water as a solvent to create an extract solution. The impact of different concentrations of these aqueous extracts on seed germination and seedling growth of the four native self-dispersed species was assessed through petri dish seed germination and pot experiments. Furthermore, a synthetical effect evaluation of the allelopathy of *Thuidium kanedae* was conducted to provide theoretical insights for the establishment, management, and upkeep of urban spontaneous plant landscapes.

## Results

### Effects of different treatments on seed germination of spontaneous plants

**Figure 1:**
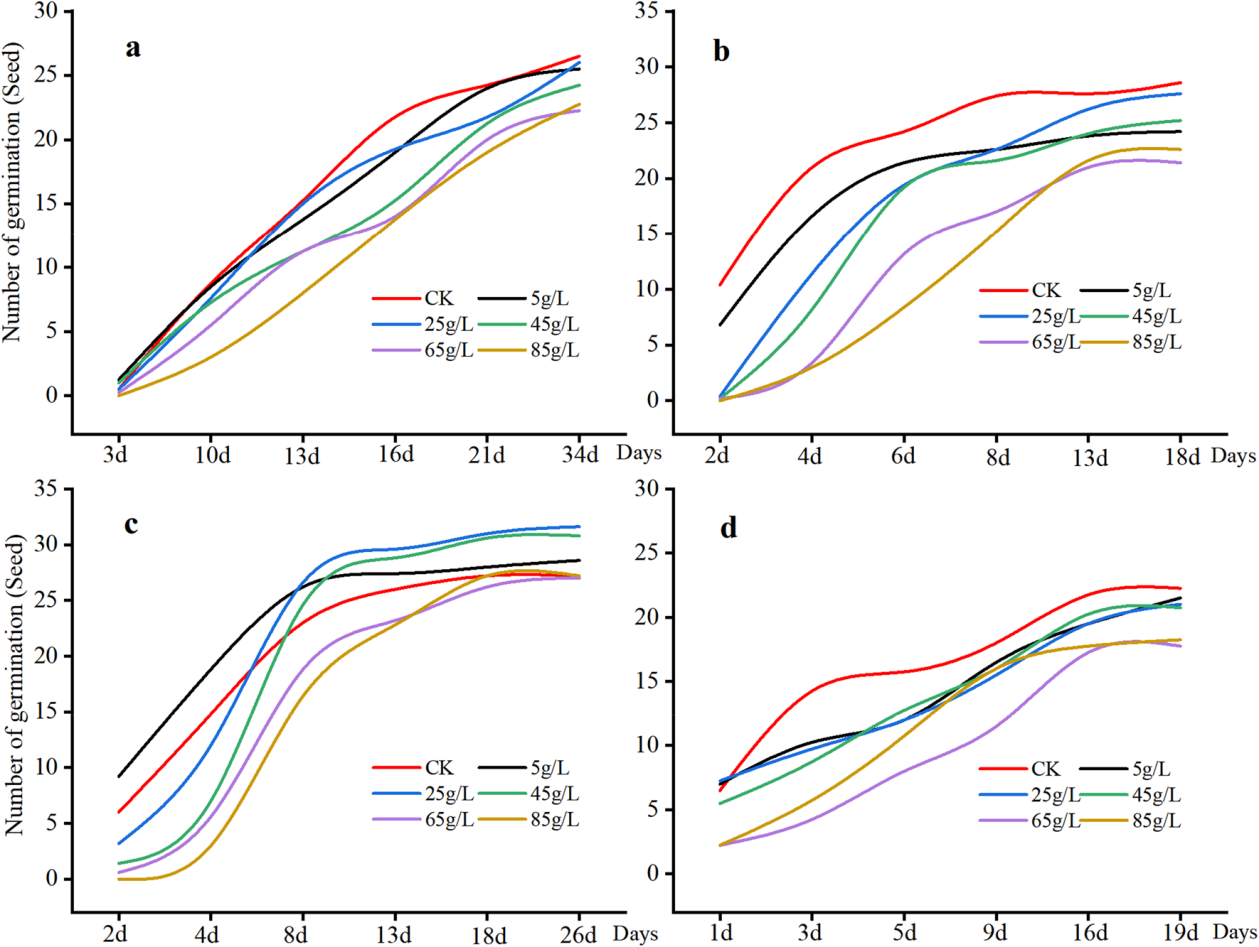
The change of seed germination number of four plant species. *Ophiopogon japonicus* (**a**), *Taraxacum mongolicum* (**b**), *Senecio scandens* (**c**), *Viola philippica* (**d**). The horizontal coordinate represents the number of days of germination. The control group was CK (red); The five concentrations of aqueous extracts were 5 g/L (black), 25 g/L (blue), 45 g/L (green), 65 g/L (purple), and 85 g/L (yellow).

The germination rates of *O. japonicus*, *T. mongolicum*, and *V. philippica* seeds treated with various concentrations of *Thuidium kanedae* were lower compared to CK (Fig. 1). Interestingly, seeds treated with concentrations ranging from 5 g/L to 45 g/L showed increasing germination rates surpassing those of the CK group after the 8th day of *S. scandens*. Additionally, the aqueous extract exhibited inhibitory effects on the germination rate, potential, and index of *O. japonicus*, *T. mongolicum*, and *V. philippica* seeds to varying extents (Table 1), with stronger inhibition observed at higher concentrations. Conversely, the aqueous extract promoted the germination rate of *S. scandens* seeds, with the potential and index displaying a pattern of "low promotion and high inhibition," indicating that lower concentrations promoted germination while higher concentrations inhibited it.

**Table 1:** Seed germination indexes of four plant species under different concentrations.

Index	Plant name	CK	5 g/L	25 g/L	45 g/L	65 g/L	85 g/L
GR	*O. japonicus*	89.17 ± 5.69a	85.84 ± 6.31a	88.34 ± 5.78a	81.67 ± 13.74ab	75.00 ± 5.77a	75.84 ± 12.58a
	*T. mongolicum*	57.20 ± 5.22a	48.40 ± 7.92bc	56.00 ± 6.00ab	50.40 ± 4.34abc	42.80 ± 3.35c	45.20 ± 7.29c
	*S. scandens*	54.40 ± 4.77a	57.20 ± 7.69a	63.20 ± 7.16a	62.00 ± 8.49a	56.00 ± 3.16a	55.20 ± 9.65a
	*V. philippica*	78.33 ± 3.34a	74.17 ± 7.88a	70.13 ± 6.76ab	69.17 ± 7.40ab	60.84 ± 8.33b	61.67 ± 10.37b
GP	*O. japonicus*	72.50 ± 5.69a	63.34 ± 8.16a	64.17 ± 7.39a	50.83 ± 8.33b	46.67 ± 6.09b	45.83 ± 6.87b
	*T. mongolicum*	48.40 ± 4.98a	42.80 ± 7.43a	38.80 ± 15.01a	38.40 ± 7.13a	26.40 ± 6.23b	16.80 ± 6.57b
	*S. scandens*	46.00 ± 4.47ab	52.40 ± 9.32a	53.20 ± 3.35a	49.20 ± 8.44a	37.60 ± 5.55bc	32.80 ± 6.87c
	*V. philippica*	57.50 ± 6.31a	48.33 ± 12.32ab	45.83 ± 5.69ab	50.00 ± 17.85ab	35.00 ± 4.30b	45.00 ± 9.63ab
GI	*O. japonicus*	4.00 ± 0.55a	3.75 ± 0.31ab	3.60 ± 0.39ab	3.32 ± 0.38bc	2.97 ± 0.20cd	2.67 ± 0.34d
	*T. mongolicum*	8.90 ± 0.31a	7.17 ± 1.20b	5.65 ± 1.51c	5.31 ± 0.86cd	4.17 ± 0.43de	3.62 ± 0.77e
	*S. scandens*	5.95 ± 0.75ab	7.15 ± 1.73a	5.92 ± 0.55ab	5.24 ± 1.06bc	4.15 ± 0.41cd	3.80 ± 0.73d
	*V. philippica*	9.13 ± 1.54a	7.44 ± 1.84ab	7.09 ± 0.77abc	6.87 ± 2.65abc	4.57 ± 1.19c	5.61 ± 0.81bc

Germination rate (GR), germination potential (GP), germination index (GI). Values in the table are the "mean ± standard deviation" of three replicates. Different lowercase letters in the same line indicated significant differences among concentrations (*P* < 0.05).

### Effects of aqueous extract on growth physiology and biochemistry of spontaneous plant seedlings.

#### Impact on seedling growth

**Figure 2:**
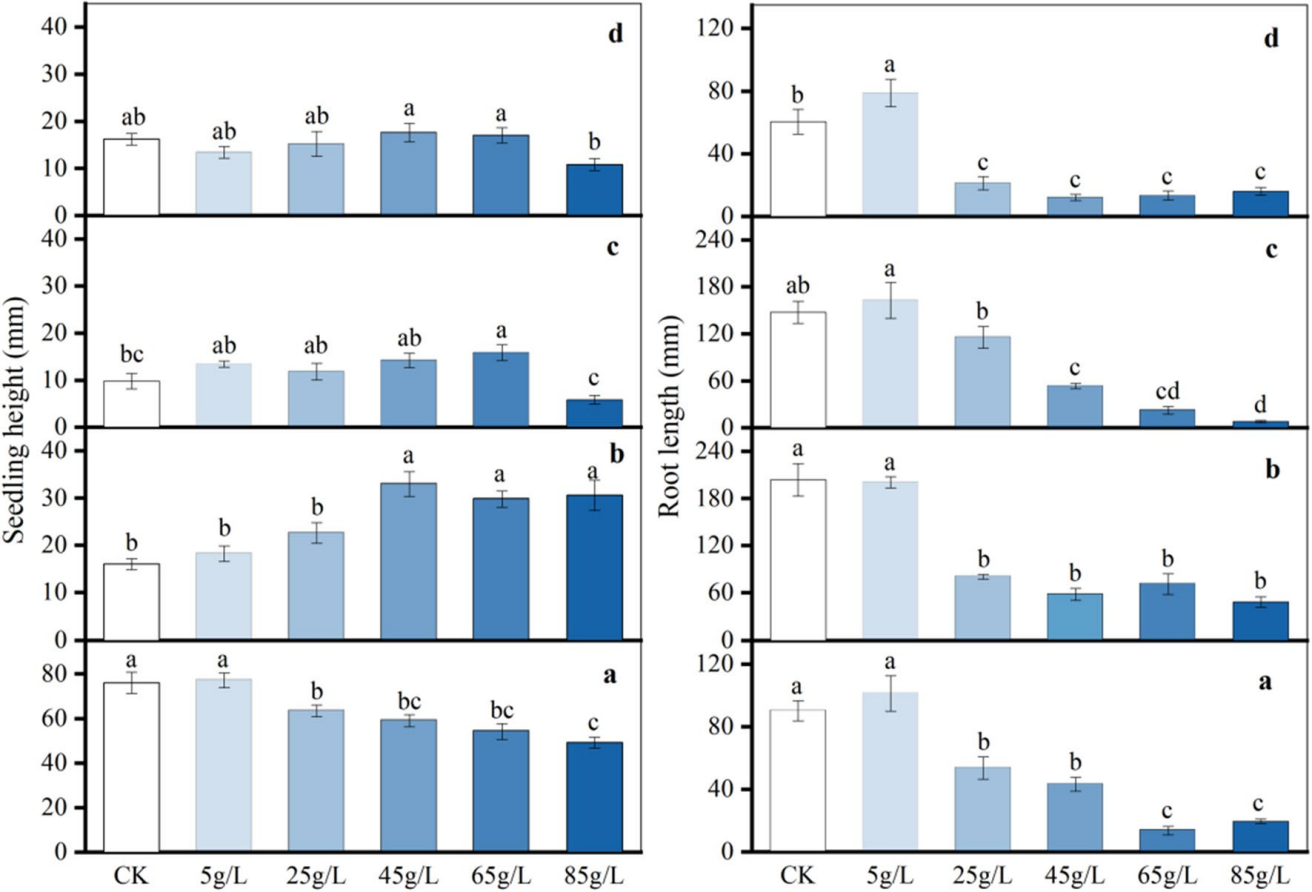
Seedling height and root length of four plant species at different concentrations. *Ophiopogon japonicus* (**a**), *Taraxacum mongolicum* (**b**), *Senecio scandens* (**c**), *Viola philippica* (**d**). Different lowercase letters of the same plant in the bars indicate significant differences between treatments (*P* < 0.05), and five replicates for each treatment.

Distinct effects on seedling height were observed with various concentrations of aqueous extract for each plant (Fig. 2, left). Over time, each concentration stimulated the growth of *T. mongolicum* seedling height, with the height at 45 g/L being 2.1 times that of the CK. Except for 5 g/L, other concentrations of the aqueous extract significantly hindered seedling height growth (*P* < 0.05).

At the lowest concentration of aqueous extract (5 g/L), root elongation in the four plant species was enhanced (Fig. 2, right). However, as the concentration increased, root elongation began to be inhibited, with a notably significant inhibitory effect observed at high concentrations (*P* < 0.01). The root length values of *T. mongolicum* and *S. scandens* in the CK were 4.3 times and 18.9 times higher than those at 85 g/L, respectively, indicating a pronounced inhibitory effect of high-concentration aqueous extract on root length.

### Effects on seedling growth and physiology

**Figure 3:**
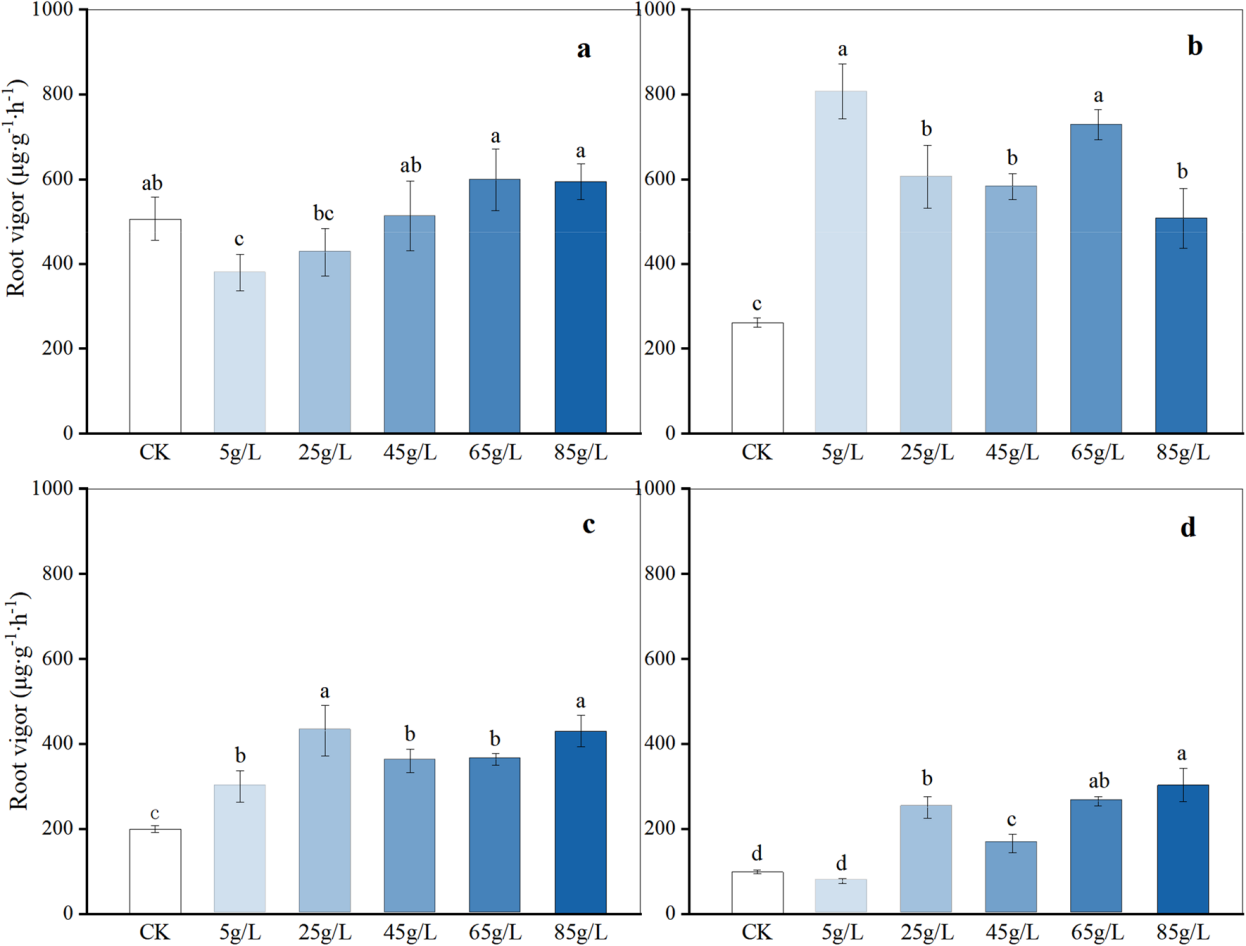
Root vigor under different concentrations of four plant species. *Ophiopogon japonicus* (**a**), *Taraxacum mongolicum* (**b**), *Senecio scandens* (**c**), *Viola philippica* (**d**). Each plate represents a plant, and the different letters on the bar within the same plate represent significant differences between treatments (*P* < 0.05).

Root vigor, encompassing the root's capacity for absorption, synthesis, oxidation, and reduction, is a crucial indicator profoundly impacting the growth and development of a plant's above-ground structures^20^. Treatment with varying concentrations of aqueous extract generally enhanced the root vigor of the four plant species (Fig. 3). Notably, the root vigor levels of *T. mongolicum* and *S. scandens* were consistently higher than those of CK across all concentrations (*P* < 0.05), with peak values reaching 3.1 times and 2.2 times that of the CK at 5 g/L and 25 g/L, respectively. Except for 5 g/L, the root vigor levels of *V. philippica* at other concentrations were also significantly elevated compared to the CK.

**Figure 4:**
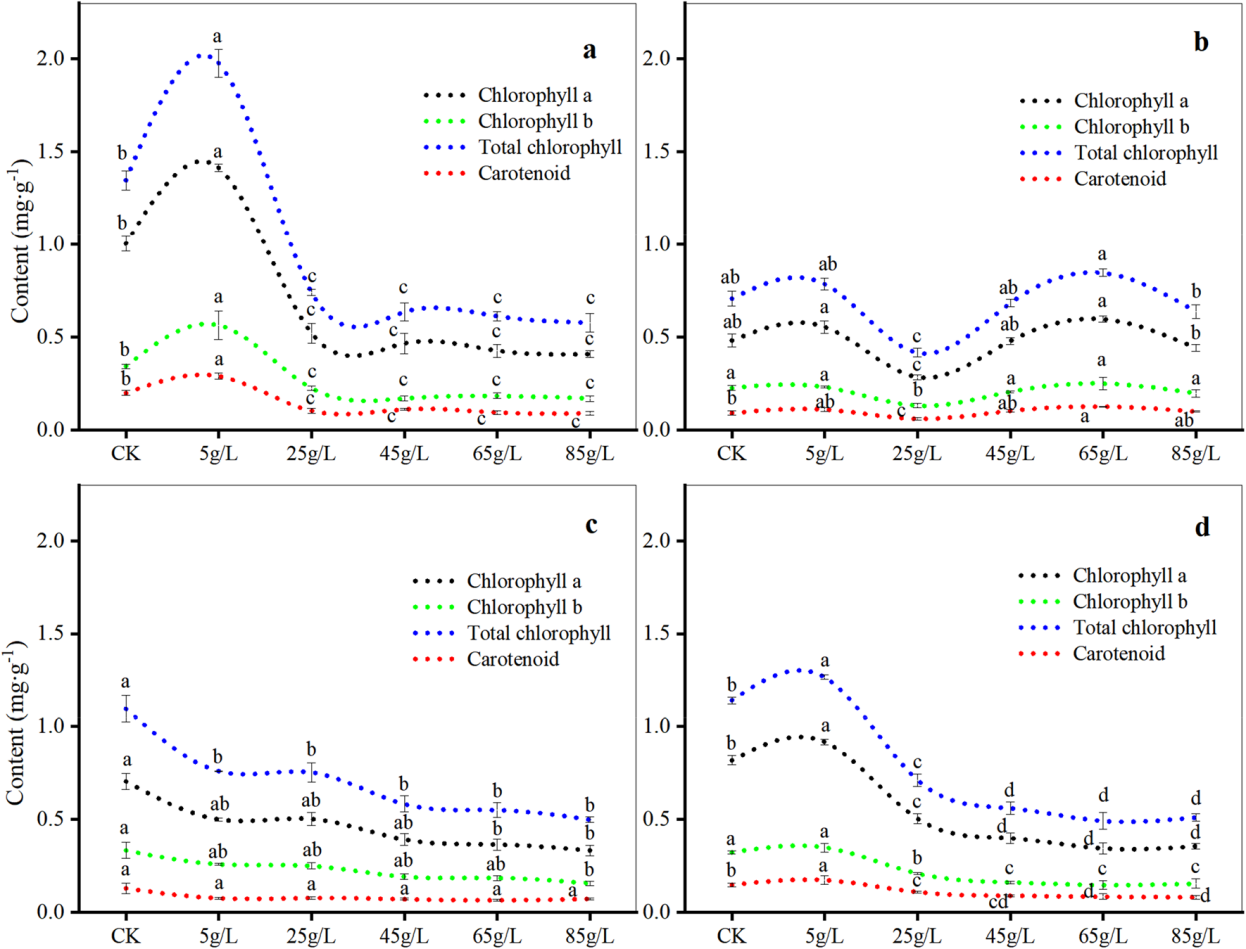
Changes of chlorophyll content of four plant species under different concentrations. *Ophiopogon japonicus* (**a**), *Taraxacum mongolicum* (**b**), *Senecio scandens* (**c**), *Viola philippica* (**d**). Chlorophyll a (black), chlorophyll b (green), total chlorophyll (blue), carotenoids (red). Different letters on the same curve represent significant differences (*P* < 0.05).

The aqueous extract had a generally suppressive effect on the chlorophyll content in the four plant species (Fig. 4), indicating its detrimental impact on the photosynthesis of their seedling leaves. Specifically, the levels of chlorophyll a, chlorophyll b, total chlorophyll, and carotenoids in *O. japonicus* and *V. philippica* were highest at 5 g/L, followed by the CK, and progressively declined with increasing concentration. *T. mongolicum* showed the lowest chlorophyll content at 25 g/L, which significantly deviated from the CK. In *S. scandens*, the chlorophyll content at each concentration was lower than that of the CK, with chlorophyll b and total chlorophyll content decreasing as the concentration increased.

### Effects on osmoregulatory substances of seedlings

**Figure 5:**
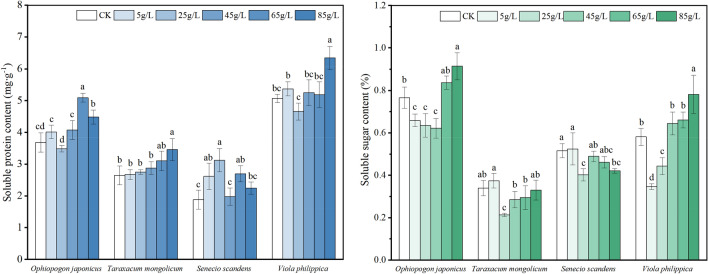
Effects of different concentrations of the aqueous extracts on osmoregulatory substances of four plant species. *Ophiopogon japonicus* (**a**), *Taraxacum mongolicum* (**b**), *Senecio scandens* (**c**), *Viola philippica* (**d**). The figure on the left shows soluble protein content, and the figure on the right shows soluble sugar content. Each plant is in a group, and different letters in the group indicate significant differences (*P* < 0.05).

The aqueous extract generally increased the content of soluble protein (SP) in the seedlings of the four plant species (Fig. 5, left). This suggests that the high concentration of the aqueous extract altered the osmotic pressure of the seedling cells, leading to increased SP production to regulate osmotic pressure. Specifically, the SP content of *T. mongolicum* seedlings gradually increased with the concentration and significantly differed from the control at 85 g/L.

The aqueous extract significantly impacted the soluble sugar (SS) content of *O. japonicus* and *V. philippica* seedlings, demonstrating a pattern of "high promotion and low inhibition," with the peak SS content recorded at 85 g/L (Fig. 5, right). In contrast, *T. mongolicum* and *S. scandens* seedlings exhibited reduced SS content compared to the CK when subjected to high-concentration treatment. Additionally, the SS content of *V. philippica* seedlings showed a gradual increase with the escalating concentration of the aqueous extract.

### Effects on membrane lipid peroxidation and enzyme activities in seedlings

**Figure 6:**
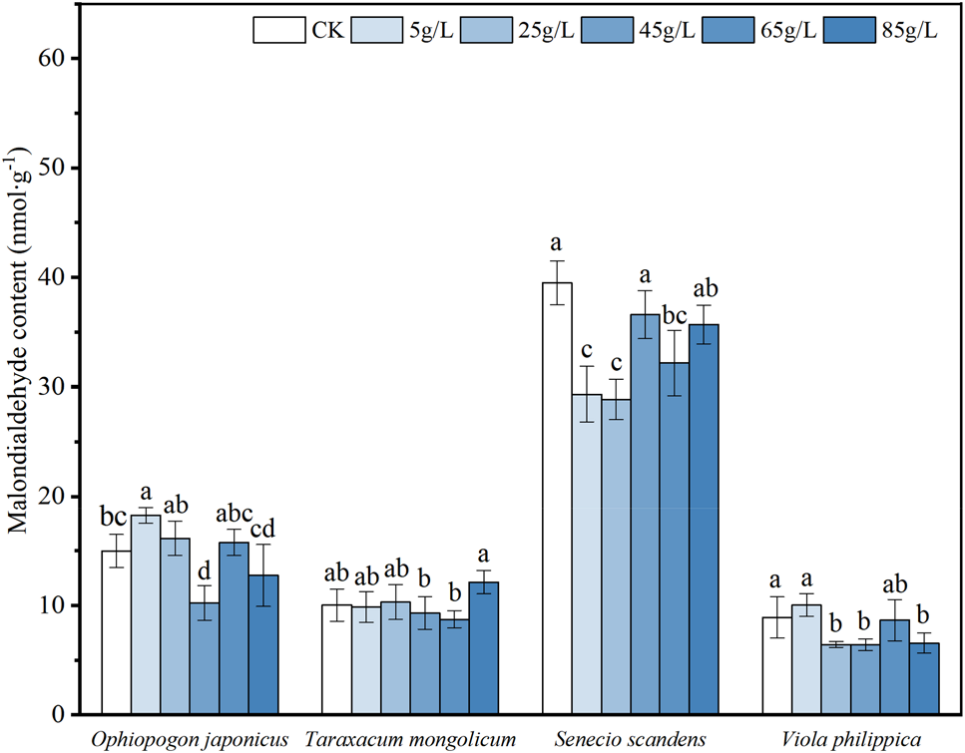
Malondialdehyde content of seedlings of four plant species under different concentrations. *Ophiopogon japonicus* (**a**), *Taraxacum mongolicum* (**b**), *Senecio scandens* (**c**), *Viola philippica* (**d**). Different letters within each plant represent significant differences (*P* < 0.05).

The high concentration of the aqueous extract generally suppressed the malondialdehyde (MDA) content of *O. japonicus*, *S. scandens*, and *V. philippica* seedlings (Fig. 6). However, it promoted the MDA content in *O. japonicus* and *V. philippica* seedlings at 5 g/L, indicating a more pronounced oxidative damage to plant membrane lipids at higher concentrations. The MDA content in *S. scandens* was lower than that of the CK at all concentrations, and similarly, the content in *V. philippica* at all concentrations except 5g/L was lower than that of the control.

**Figure 7:**
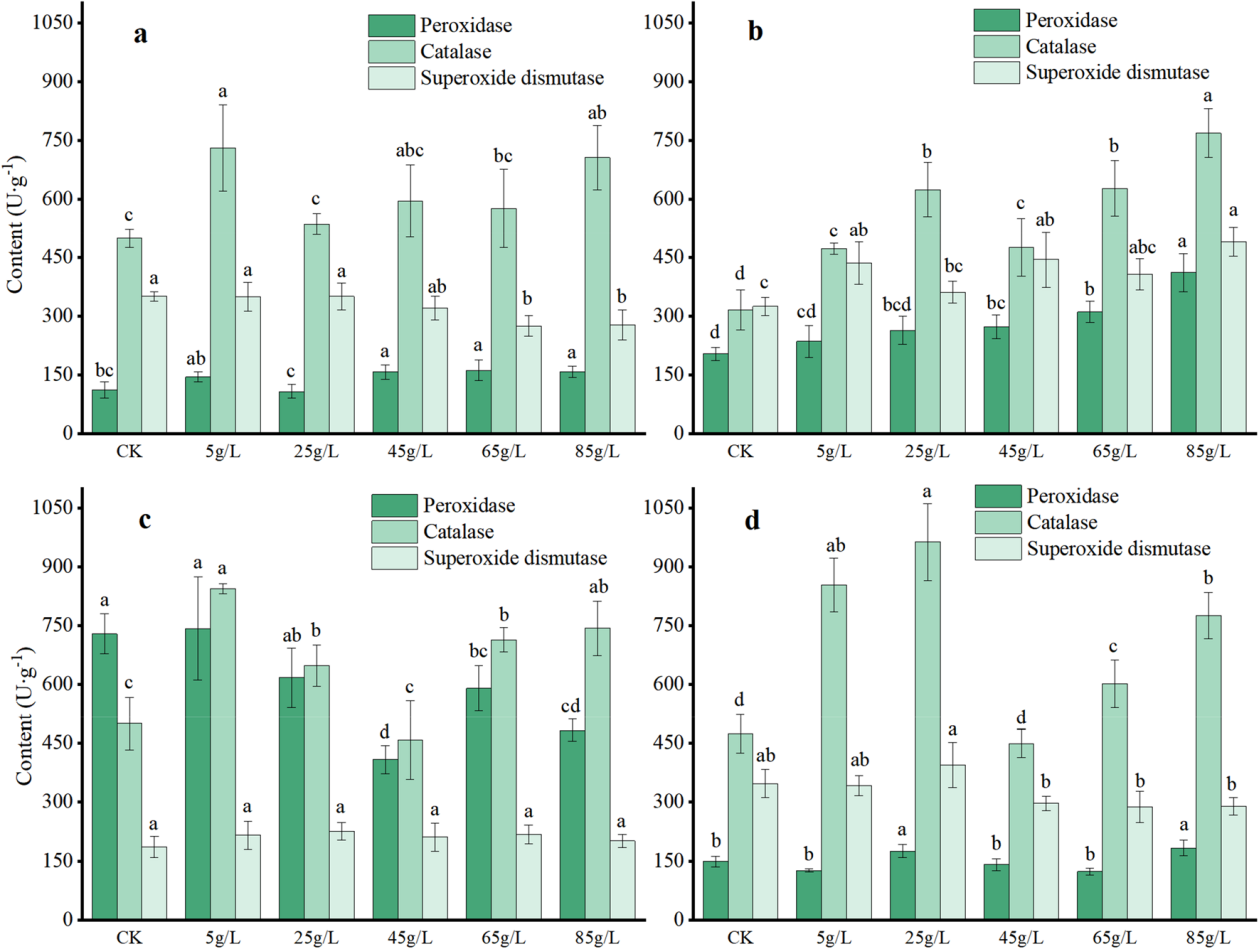
Effects of different concentrations of the aqueous extracts on enzyme activity of four plant species. *Ophiopogon japonicus* (**a**), *Taraxacum mongolicum* (**b**), *Senecio scandens* (**c**), *Viola philippica* (**d**). Each plate represents a plant, and the same color represents the amount of an enzyme. Different letters on columns of the same color within each plate represent significant differences (*P* < 0.05).

The treatment with the aqueous extract enhanced the activities of peroxidase (POD) and catalase (CAT) in *O. japonicus* seedlings, as well as the activities of POD, CAT, and superoxide dismutase (SOD) in *T. mongolicum*. Additionally, it increased the CAT activities of *S. scandens* and *V. philippica*, but inhibited the activities of SOD in *O. japonicus* and *S. scandens* seedlings (Fig. 7). Notably, the POD and CAT activities of *T. mongolicum* seedlings peaked at 85 g/L, reaching levels 2 times and 2.5 times that of the CK, respectively. These results indicate that the aqueous extract imposed a certain level of stress on the growth of the four plant species, leading to increased enzyme activity in the seedlings' antioxidant system to mitigate adverse effects.

### Evaluation of allelopathic comprehensive effect

Based on the allelopathy synthetical effect (SE) of seed germination (Table 2), the aqueous extract demonstrated an allelopathic inhibition effect on the seed germination of *O. japonicus*, *T. mongolicum*, and *V. philippica* (SE < 0). In terms of seedling growth morphology, four plant species exhibited an allelopathic promotion effect at the lowest concentration of 5 g/L (SE > 0), while higher concentrations showed an allelopathic inhibition effect, with a more pronounced inhibition effect at elevated concentrations. This indicates that the aqueous extract negatively affected the morphological growth of the seedlings. Analysis of seedling physiology revealed that the five concentrations of SE in *T. mongolicum* and *S. scandens* displayed allelopathic promotion, while the other treatments except 5 g/L in *O. japonicus* were allelopathic inhibition. The patterns governing the SE concerning osmotic regulatory substances, MDA, and enzyme activity index varied among the four plant species. In summary, the aqueous extract facilitated the regulation of osmotic pressure changes in the four plant species by accumulating osmotic substances and enhancing enzyme activity to support normal growth. Based on the physiological and biochemical SE, the aqueous extract promoted the physiological and biochemical aspects of *T. mongolicum*, *S. scandens*, and *V. philippica*, while inhibiting *O. japonicus* seedlings.

**Table 2:** The synthetical effect (SE) of allelopathy index of four plant species in each index stratum.

Plant name	Index level	Concentration of aqueous extract					SE
		5g/L	25g/L	45g/L	65g/L	85g/L	
*O. japonicus*	Seed germination	-0.076	-0.075	-0.184	-0.258	-0.283	-0.175
	Seedling growth morphology	0.062	-0.286	-0.372	-0.568	-0.568	-0.346
	Seedling physiology	0.082	-0.297	-0.236	-0.185	-0.263	-0.180
	Osmoregulatory substance	-0.025	-0.112	-0.054	0.179	0.170	0.032
	MDA and enzyme activity	0.279	-0.107	-0.076	0.029	-0.091	0.007
	Physiology and biochemistry	0.104	-0.203	-0.150	-0.041	-0.112	-0.080
*T. mongolicum*	Seed germination	-0.154	-0.195	-0.243	-0.412	-0.485	-0.298
	Seedling growth morphology	0.053	-0.157	-0.102	-0.095	-0.145	-0.089
	Seedling physiology	0.391	0.093	0.279	0.412	0.246	0.284
	Osmoregulatory substance	0.047	-0.172	-0.040	0.001	0.094	-0.014
	MDA and enzyme activity	0.069	0.112	0.073	0.073	0.254	0.116
	Physiology and biochemistry	0.224	0.032	0.148	0.224	0.210	0.168
*S. scandens*	Seed germination	0.113	0.090	0.023	-0.152	-0.211	-0.027
	Seedling growth morphology	0.181	-0.023	-0.165	-0.235	-0.678	-0.184
	Seedling physiology	0.116	0.182	0.089	0.074	0.096	0.112
	Osmoregulatory substance	0.152	0.051	0.018	0.104	-0.004	0.064
	MDA and enzyme activity	-0.022	-0.065	-0.094	-0.015	-0.026	-0.045
	Physiology and biochemistry	0.091	0.088	0.025	0.059	0.041	0.061
*V. philippica*	Seed germination	-0.133	-0.181	-0.161	-0.372	-0.272	-0.224
	Seedling growth morphology	0.030	-0.355	-0.361	-0.367	-0.534	-0.317
	Seedling physiology	-0.056	0.104	-0.032	0.108	0.113	0.047
	Osmoregulatory substance	-0.174	-0.160	0.099	-0.009	0.225	-0.004
	MDA and enzyme activity	0.040	0.017	-0.189	-0.078	0.003	-0.041
	Physiology and biochemistry	-0.061	0.016	-0.038	0.032	0.113	0.012

### Allelopathy verification of the two substances

**Figure 8:**
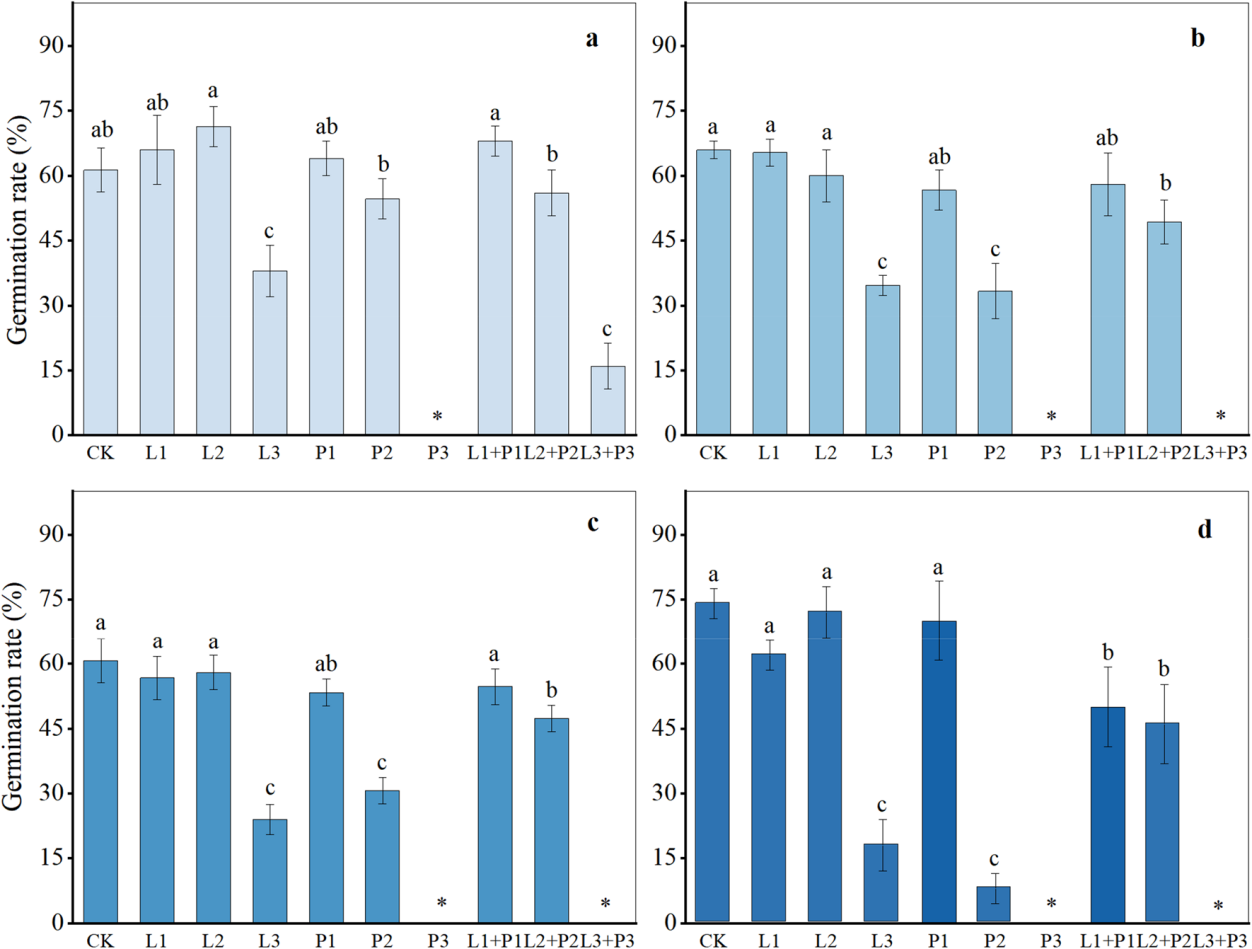
Effect of different concentrations on seed germination rate of four plant species. *Ophiopogon japonicus* (**a**), *Taraxacum mongolicum* (**b**), *Senecio scandens* (**c**), *Viola philippica* (**d**). * indicates that none of the seeds in this treatment germinated, resulting in a germination rate of 0. CK represents distilled water; L1, L2, L3 represent concentrations of 0.1 mg/mL, 1 mg/mL, 10 mg/mL of L-phenylalanine solution, respectively; P1, P2, P3 represent concentrations of 0.1 mg/mL, 1 mg/mL, 10 mg/mL of 2-phenylethanol solution, respectively; L1 + P1, L2 + P2, and L3 + P3 represent mixed solutions of 0.1 mg/mL, 1 mg/mL, and 10 mg/mL, respectively. Different letters on each bar represent significant differences (*P* < 0.05).

The verification experiment indicated that solutions containing L-phenylalanine, 2-phenylethanol, and their mixtures significantly inhibited the seed germination of *T. mongolicum*, *S. scandens*, and *V. philippica* (*P* < 0.01) (Fig. 8). Under the P3 and L3 + P3 treatments, the seeds of these three plants failed to germinate, suggesting inhibition or complete lack of germination at high concentrations. The seed of *O. japonicus* exhibited the highest germination rate under the L2 treatment, with higher rates observed under L2, L1 + P1, L1, and P1 treatments compared to CK, indicating that lower solution concentrations could promote seed germination of *O. japonicus*. Moreover, the germination rates of the four plant species treated with the 2-phenylethanol solution were generally lower, indicating a stronger inhibitory effect compared to the L-phenylalanine solution and the mixed solution of both. In conclusion, considering the growth and physiological responses of the four plant species alongside the content of the two substances in the aqueous extract, it can be inferred that both 2-phenylethanol and L-phenylalanine exert potent allelopathy on the four plant species, with 2-phenylethanol demonstrating stronger allelopathic activity.

## Discussion

Allelopathy primarily influences seed germination and seedling growth, as documented in the literature^21,22^. It is widely reported that allelopathic interactions can result in "low promotion and high inhibition" effects on seed germination of recipient plants^23,24^, although some studies have reported exclusively inhibitory outcomes^25–28^. In our study, the aqueous extract of *T. kanedae* markedly suppressed the germination of *O. japonicus*, *T. mongolicum*, and *V. philippica* seeds, with the degree of inhibition intensifying at higher concentrations. This suggests a correlation between the concentration of the aqueous extract and the levels of allelochemicals, leading to a more pronounced inhibitory effect. The suppression of seed germination by the aqueous extract may be attributed to the allelochemicals interfering with the metabolic pathways during seed embryo growth and germination, disrupting the balance of endogenous hormones, and consequently diminishing seed viability and preventing germination^29,30^. The response of *S. scandens* seeds to the aqueous extract exhibited a "low promotion and high inhibition" pattern, which could be due to the species' varying tolerance to the extract. At lower concentrations, the effect might be negligible or even stimulatory, potentially due to the selectivity of allelochemicals. However, as seedlings developed, the influence of the aqueous extract on germination diminished or became inhibitory, which might be associated with the increased resistance of *S. scandens* during the seedling stage^31^.

Allelopathy also exerts a significant influence on the growth physiology of plant seedlings. Shan Guilian et al. reported that crushed *Euphorbia jolkinii* Boiss. plants led to diminished root vigor, increased activity of antioxidant enzymes, and inhibited chlorophyll synthesis in *Medicago sativa* L., thereby suppressing seedling growth^32^. He Deng et al. demonstrated that a combination of high-concentration *Eucalyptus robusta* Sm. leaf aqueous extract and low-concentration wood vinegar notably enhanced root elongation^33^. In our study, we observed that the lowest concentration (5 g/L) of *T. kanedae* aqueous extract acted as a root length promoter for the four examined plants. However, higher concentrations of the extract significantly impeded root elongation, aligning with findings by Magdalena Lenda et al.^34^. The allelochemicals present in the aqueous extract disrupted cellular membrane integrity, inhibited apical cell division, and curtailed the growth of embryonic roots and axes^35–37^, which collectively obstructed root system elongation and led to a reduction in root length. Despite constraining root elongation, the aqueous extract notably increased root vigor in *T. mongolicum* and *S. scandens* and generally enhanced root vigor in *O. japonicus* and *V. philippica*. This could be attributed to the extract supplying nutrients that bolstered root system uptake.

Allelochemicals have the potential to influence plant photosynthesis by altering chlorophyll levels^38^. Our study observed that, at a concentration of 5 g/L, the chlorophyll content in the leaves of *O. japonicus* and *V. philippica* was increased. This increase could be attributed to the relatively mild allelopathic inhibition at lower concentrations. However, concentrations exceeding 5 g/L showed inhibitory effects, leading to reduced chlorophyll content compared to the CK. Additionally, the aqueous extracts suppressed the chlorophyll content of *S. scandens*, with the inhibitory effect becoming more pronounced with higher concentrations, in line with the findings of Niu Huanhuan et al.^39^. In conclusion, the aqueous extract had a negative impact on the photosynthesis of *O. japonicus*, *S. scandens*, and *V. philippica*, hindering the production of the photosynthetic pigment chlorophyll and consequently adversely affecting the growth of these three plants.

Allelochemicals can induce elevated levels of reactive oxygen species (ROS) within plant cells, prompting the activation of antioxidant defense systems to mitigate oxidative damage^40,41^. Superoxide dismutase (SOD), catalase (CAT), and peroxidase (POD) are pivotal enzymes that regulate ROS accumulation in plants. These enzymes work synergistically to reduce cellular damage from ROS-induced lipid peroxidation of membranes^42^. SOD activity is a direct indicator of a plant's resistance to adverse environmental conditions, while POD and CAT activities reflect the extent of damage sustained by the plant^43^. In our study, POD activity in *O. japonicus* and *T. mongolicum* seedlings increased with higher concentrations of the aqueous extract, and CAT activity in all four plant species also exhibited a general upward trend. This suggests that the plants experienced allelopathic stress from the extract, leading to excessive ROS production and a consequent rise in antioxidant enzyme activity to maintain essential physiological metabolism, aligning with findings by Hu Huan et al.^44^. However, the capacity of the antioxidant enzyme system to regulate is temporary and finite. Once oxidized products accumulate beyond a certain threshold, various enzymes may become impaired, resulting in decreased activity^45^. The variation in antioxidant enzyme content among the four plant species in our study likely reflects their distinct regulatory capacities and tolerance levels, thereby manifesting different allelopathic responses.

Despite their simple structure, lack of root, stem, and leaf differentiation, and absence of true vascular tissue, bryophytes demonstrate a remarkable ability to thrive and flourish in diverse environments, resulting in the production of a wide array of rich and diverse secondary active components^46^. Among them, phenolic acids and terpenoids are recognized for their allelopathic effects^47^. Secondary metabolites originating from bryophytes mainly include terpenes, phenols, lipids, sterols, aromatic compounds, and more. Terpenes encompass monoterpenes, sesquiterpenes, diterpenes, and triterpenes, while phenolic compounds consist of derivatives of benzoic acid, cinnamic acid, flavonoids, and other phenolic substances^48^. For example, Hisashi et al. identified the allelopathic active substances Momilactone A and B, as well as the primary allelopathic substance 3-hydroxy-β-porphyrodone in *Brachyglossum japonica*^49^. Li et al. isolated trichoderolactone F, a novel carotene-type diterpenoid compound, and identified 17 known compounds through chemical analysis of secondary metabolites from moss. Among them, momilactone F, acrenol, momilactones A and B exhibited significant allelopathic activity against *Samolus parviflorus* Raf. and *Lactuca sativa* L. var. *angustana Irish*^50^. Our study unveiled that the aqueous extract contains a high content of various allelochemicals such as phenols, alkaloids, flavonoids, terpenoids, including L-phenylalanine, 2-phenylethanol, oleic acid, palmitic acid, and more. The secondary metabolic substances of *T. kanedae* encompasses a wide range of allelochemicals and potential allelopathic activity.

Allelochemicals exhibit selectivity and specificity^51^. Prior research has identified that inhibitors of seed germination are predominantly water-soluble organic acids, alcohols, and phenols^52,53^. Among these, phenolic compounds are quintessential allelochemicals, ubiquitously present in plants. They can impede water and nutrient uptake by roots and hinder plant growth by affecting photosynthesis and enzyme activities^54,55^. Numerous studies have documented the allelopathic impact of phenolics. In our study, the aqueous extract displayed the second-highest content of phenolic substances, with 2-phenylethanol identified as the most abundant compound. Our verification experiment revealed that 2-phenylethanol significantly inhibited seed germination, as none of the four plant species germinated when exposed to a 10 mg/mL solution of 2-phenylethanol, whereas seeds treated with a similar concentration of L-phenylalanine did germinate. These results indicate that the allelopathic activity of 2-phenylethanol exceeds that of L-phenylalanine, highlighting the potent allelopathic effects of phenolic substances. Interestingly, L-phenylalanine, the primary compound in the aqueous extract, demonstrated inhibitory effects on germination at high concentrations but promoted germination at lower concentrations. This dual effect may be due to the substance's weak allelopathic activity at low concentrations or its potential role as a seed nutrient, thereby facilitating germination. Additionally, although amino acids and their derivatives were the most prevalent substances in the aqueous extract, other compounds were present in smaller amounts. Further research is needed to elucidate the specific functions of these substances and their underlying mechanisms of action.

## Conclusion

Our study represents the first investigation into the allelopathic effects of *Thuidium kanedae* on its neighboring symbiotic flora and verifies the allelopathic effects of substances present in the aqueous extract. The findings revealed that the aqueous extract of *T. kanedae* significantly suppressed the seed germination of *O. japonicus*, *T. mongolicum*, and *V. philippica*, while its impact on *S. scandens* germination showed a pattern of promotion at minimal concentrations and inhibition at higher concentrations. The aqueous extract promoted the growth of the four plant species only at the lowest concentration, and inhibited the rest concentration. Physiological and biochemical effects on *O. japonicus* seedlings were mainly inhibitory, whereas effects on *T. mongolicum* and *S. scandens* seedlings were promotive. Particularly, the allelopathic influence of 2-phenylethanol and L-phenylalanine was more pronounced in these species, with 2-phenylethanol exhibiting stronger allelopathic activity. Consequently, *T. kanedae* exerts allelopathic effects on neighboring spontaneous plants, with the extent of influence varying among different species. In green spaces where *T. kanedae* is sparsely distributed, its inhibitory effect is diminished or may even enhance growth, suggesting that in bryophyte-dominated habitats, the seeds of spontaneous plants can selectively germinate and grow, contributing to the formation of a distinctive wilderness landscape. The evolution of such landscapes may be influenced by the density of the bryophyte population. The findings of this study provide new insights for the management and utilization of spontaneous plants, serving as a theoretical foundation for the development and maintenance of urban spontaneous plant landscapes and enhancing the diversity of plant species within urban landscape.

## Materials and methods

### Plant materials

The donor bryophytes were chosen based on their prevalence, ornamental value, wide distribution, abundance in growing patches, and frequent cohabitation with other plants in the green spaces of Guiyang City. The selection of recipient spontaneous plants involved field surveys, literature reviews, content analysis, questionnaire surveys, consideration of ornamental value, ecological benefits, development and utilization potential of the plants, as well as expert consultations, resulting in the comprehensive selection of representative herbaceous spontaneous plants.Here are the plants we selected: *Thuidium kanedae*, is a member of the Thuidiaceae family and was sourced from Guanshanhu Park in Guiyang City, Guizhou Province, China. We procured seeds of four spontaneous plant species from online sources, namely: *Ophiopogon japonicus* (L. f.) Ker Gawl. (Asparagaceae); *Taraxacum mongolicum* Hand.-Mazz. (Asteraceae); *Senecio* *scandens* Buch.-Ham. ex D. Don (Asteraceae); and *Viola philippica* Cav. (Violaceae).

### Preparation of aqueous extract

First, remove impurities from fresh *T. kanedae*, rinse it with tap water and distilled water, and allow it to naturally dry. Then, dry the moisture in an oven at 70 °C, cut it into pieces, and pulverize it into powder. Subsequently, place 10 g of the powder into a conical bottle, add 100 ml of distilled water, seal the bottle with a sealing film, and soak it at room temperature for 24 h. Next, place it into a shaker at 35 °C and 180 r/min for constant temperature oscillation for 12 h. Finally, filter the filtrate using gauze to obtain a concentration of 100 g/L. Based on the concentration setting, different volumes of distilled water were added to dilute, resulting in aqueous extracts of *T. kanedae* with concentrations of 5 g/L, 25 g/L, 45 g/L, 65 g/L, and 85 g/L. The distilled water was used as control (CK) and stored in the refrigerator at 4 °C for later use.

### Seed germination experiment and index determination

The seeds of four plant species were disinfected with a 1% sodium hypochlorite solution for 30 min, then washed and set aside. In each petri dish with a 10 cm diameter, two pieces of filter paper were placed at the bottom. An equal number of healthy, uniform-sized seeds (30 seeds each of *O. japonicus* and *V. philippica*, 50 seeds each of *T. mongolicum* and *S. scandens*) were evenly distributed in the dishes. Subsequently, 4 ml of aqueous extract at varying concentrations were added to each dish (with distilled water serving as the control), and the petri dishes were then placed in an intelligent artificial climate chamber. The climate chamber conditions were set as follows: 12 h of light, 3000lx intensity, 25 °C temperature, and 70% humidity during the day, and 12 h of darkness, 20 °C temperature, and 75% humidity at night^56^. The germination situation was observed and recorded daily. Germination is considered to be over when no new seeds germinate within 3 consecutive days. From the recorded germination numbers, the germination rate^57^, germination potential^58^, and germination index^59^ were calculated using the following formulas:

1 $${\text{Germination}}\;{\text{rate}}({\text{GR}}) = \left( {{\text{total}}\;{\text{number}}\;{\text{of}}\;{\text{normal}}\;{\text{germinated}}\;{\text{seeds}}/{\text{total}}\;{\text{number}}\,{\text{of}}\;{\text{tested}}\;{\text{seeds}}} \right) \times 100\%$$

2 $${\text{Germinating}}\;{\text{potential}}\,({\text{GP}}) = \left( {{\text{number}}\;{\text{of}}\,{\text{germinations}}\;{\text{in}}\;{\text{the}}\;{\text{first}}\;2/3\;{\text{of}}\;{\text{the}}\;{\text{germination}}\;{\text{cyclicality}}/{\text{total}}\;{\text{number}}\;{\text{of}}\;{\text{seeds}}\;{\text{tested}}} \right) \times 100\%$$

3 $${\text{Germination}}\;{\text{index}}\left( {{\text{GI}}} \right) = \sum Gt/Dt$$

where Gt is the germination number of seeds on the t day, and Dt is the corresponding germination days.

After the germination test, five seedlings were randomly selected from each concentration, and their root length and seedling height were measured using a ruler^60^. The root vigor of seedlings was determined using the 2,3,5-triphenyltetrazolium chloride (TTC) method^61^. The chlorophyll content was determined using the 95% ethanol extraction method, and the contents of chlorophyll a, chlorophyll b, carotenoids, and total chlorophyll were calculated according to the Arnon method^62^.

### Seedling experiment and index determination

The seedling experiment was conducted at the experimental base of the Forestry College of Guizhou University (106°65′ ~ 107°17′E, 26°45′ ~ 27°22′N) in Huaxi District, Guiyang City, Guizhou Province, China. The location has a subtropical humid-moderate climate, with an altitude of 1137.76 m, an average annual temperature of 15.3 °C, and an average total annual precipitation of 1129.5 mm. Greenhouses with two layers of 4-needle sunshade nets were used for shading treatment (shading rate of 65% ~ 75%). Each plastic pot for planting (outer diameter 14 cm, height 12 cm, bottom diameter 9.2 cm) was filled with an equal amount of Chuangyao No.1 soil from Guizhou Chuangyao Agricultural Biotechnology Co., Ltd. Eight seedlings cultivated in the incubator were planted in each pot. A 50 mL *T. kanedae* aqueous extract of different concentrations (distilled water for the control) was applied once a week, with regular watering management at other times. There were 15 replicates per treatment, and the experiment lasted for 120 days. The physiological indexes of the seedlings were measured at the end of the experiment.

The determination of physiological indexes of seedlings involved measuring the soluble sugar (SS) content using the anthrone colorimetric method and the soluble protein (SP) content using the Caulmers Brilliant Blue G-250 (Bradford) method^63^. Malondialdehyde (MDA), peroxidase (POD), catalase (CAT), and superoxide dismutase (SOD) were determined using kits purchased from Guizhou Ruigewai Technology Co., Ltd., under the brand Grace.

### Detection of Substance Composition in Aqueous Extract and Verification of Allelopathy

The composition of substances in the aqueous extract of *T. kanedae* was analyzed by Shanghai Baiqu Biomedical Technology Co., Ltd. The chromatographic analysis involved separating target compounds using a Waters UPLC liquid chromatographic column within an EXION LC System (SCIEX) ultra-high performance liquid chromatograph. The liquid chromatography utilized a 0.1% formic acid aqueous solution as phase A and acetonitrile as phase B. The column temperature was maintained at 40 °C, the automatic sampler temperature at 4 °C, and a sample volume of 2 μL was injected. Mass spectrometry was performed using a SCIEX 6500 QTRAP + triple quadrupole mass spectrometer equipped with an IonDrive Turbo V ESI ion source operating in Multiple Reaction Monitoring (MRM) mode. Data acquisition and quantitative analysis of the target compounds were carried out using SCIEX Analyst Work Station Software (Version 1.6.3).

The two substances with the highest relative content in the aqueous extract, L-phenylalanine and 2-phenylethanol, were selected for the allelopathy verification test (Table 3). These substances were obtained from Guizhou Green Biotechnology Co., Ltd. and prepared into solutions of varying concentrations (Table 4). A seed germination experiment was conducted using the prepared solutions on the four plant species under study. The experimental procedures and germination conditions remained consistent with previous tests. Observations were made on the germination status, recorded, and germination rates were calculated.

**Table 3:** Main substances and contents in the aqueous extract of *Thuidium kanedae*.

No	Compound name	Molecular formula	Class	Relative content(%)
1	L-Phenylalanine	C_9_H_11_NO_2_	Amino acid and derivatives	24.32
2	2-Phenylethanol	C_8_H_10_O	Phenols	16.52
3	4-Hydroxyphenylacetylgl-utamic acid	C1_3_H_15_NO_6_	Carboxylic acids and derivatives	6.98
4	Styrene	C_8_H_8_	Benzene and substituted derivatives	3.88
5	Piperlonguminine	C_16_H_19_NO_3_	Alkaloids	3.12
6	5-Aminovaleric acid	C_5_H_11_NO_2_	Amino acid and derivatives	2.82
7	L-Valine	C_5_H_11_NO_2_	Amino acid and derivatives	2.72
8	Nicotinic acid	C_6_H_5_NO_2_	Nicotinic acid derivatives	2.64
9	Oleic acid	C_18_H_34_O_2_	Fatty Acyls	2.38
10	Miltirone	C_19_H_22_O_2_	Diterpenoids	2.29
11	Phloretic acid	C_9_H_10_O_3_	Phenols	2.10
12	D-alpha-Aminobutyric acid	C_4_H_9_NO_2_	Carboxylic acids and derivatives	1.64
13	6-Aminocaproic acid	C_6_H_13_NO_2_	Fatty Acyls	1.53
14	L-Pipecolic acid	C_6_H_11_NO_2_	Amino acid and derivatives	1.12

**Table 4:** Configuration of solutions of the two substances.

Compound	Different concentrations of solution(mg/mL)	Definition
L-Phenylalanine	0.1 mg/mL	L1
	1 mg/mL	L2
	10 mg/mL	L3
2-Phenylethanol	0.1 mg/mL	P1
	1 mg/mL	P2
	10 mg/mL	P3
Mixture of L-Phenylalanine and 2-Phenylethanol	0.1 mg/mL	L1 + P1
	1 mg/mL	L2 + P2
	10 mg/mL	L3 + P3
Distilled water		CK

### Allelopathy evaluation and data analysis

The allelopathy of four plant species was assessed using the allelopathy index^64^ and the combined allelopathic effect^65^. The allelopathic effect response index (RI) was calculated using the formula:

4 $${\text{RI}} = 1 - {\text{C}}/{\text{T }}\left( {{\text{T}} \ge {\text{C}}} \right)\;{\text{or}}\;{\text{RI}} = {\text{T}}/{\text{C}} - 1\left( {{\text{T}} < {\text{C}}} \right)$$

where C represents the control value and T represents the treatment value. A positive RI value indicates a promotion of allelopathy, while a negative value indicates an inhibitory effect. The absolute value signifies the intensity of the action. the synthetical effect (SE) of allelopathy index reflects the strength of the allelopathic effect, representing the arithmetic mean value of each test index RI for the same receptor. The measured indexes in our study were categorized into five groups: seed germination (GR, GP, and GI), seedling growth morphology (seedling height and root length), seedling physiology (root vigor, chlorophyll a, chlorophyll b, and carotenoids), osmoregulatory substances (SS and SP), MDA, and enzyme activities (POD, CAT, and SOD). The SE for each corresponding index layer at five concentrations was calculated.

All experimental data were statistically organized using WPS Office tables. IBM SPSS Statistics 21 software was employed for one-way ANOVA, with a significance level set at 0.05. Duncan's multiple comparison was conducted using Origin21.0 mapping software.

### Plant ethics statement

In our experimental research/collection of plant/plant material, we strictly comply with the relevant institutional, national and international guidelines and legislation to ensure that our activities comply with the requirements of plant ethics.

## Data Availability

All data are presented in the article, and can be requested from the corresponding author if required.
